# Recent advances in managing idiopathic pulmonary fibrosis

**DOI:** 10.12688/f1000research.10720.1

**Published:** 2017-11-27

**Authors:** Chiara Scelfo, Antonella Caminati, Sergio Harari

**Affiliations:** 1 Unità Operativa di Pneumologia e Terapia Semi-Intensiva Respiratoria, Servizio di Fisiopatologia Respiratoria ed Emodinamica Polmonare, Ospedale San Giuseppe, Multimedica IRCCS, Milan, Italy

**Keywords:** idiopathic pulmonary fibrosis, pirfenidone, nintedanib, pathogenesis

## Abstract

Idiopathic pulmonary fibrosis (IPF) is a rare pulmonary disease with a poor prognosis and severe impact on quality of life. Early diagnosis is still challenging and important delays are registered before final diagnosis can be reached. Available tools fail to predict the variable course of the disease and to evaluate response to antifibrotic drugs. Despite the recent approval of pirfenidone and nintedanib, significant challenges remain to improve prognosis and quality of life. It is hoped that the new insights gained in pathobiology in the last few years will lead to further advances in the diagnosis and management of IPF. Currently, early diagnosis and prompt initiation of treatments reducing lung function loss offer the best hope for improved outcomes. This article aims at providing an overview of recent advances in managing patients with IPF and has a particular focus on how to reach a diagnosis, manage comorbidities and lung transplantation, care for the non-pharmacological needs of patients, and address palliative care.

## Introduction

Idiopathic pulmonary fibrosis (IPF) is a progressive fibrosing disease of unknown cause limited to the lungs. It is a fatal, age-related lung disease characterized by a mean survival time ranging from 3 to 5 years ^1^. In Europe and North America, the incidence of IPF is 3–9 cases per 100,000 people and is increasing worldwide ^2, 3^.

Knowledge about IPF pathogenesis is evolving and the currently prevailing hypothesis is that the disease involves a crosstalk between the alveolar epithelium and underlying mesenchyme leading to aberrant wound healing, scarring of the lung, and progressive loss of function ^1^. Despite recent advances in understanding the disease pathobiology, IPF management remains difficult, particularly because of its unpredictable course with some patients experiencing prolonged periods of slow and progressive decline and others succumbing to acute exacerbations (AEs).

This article aims at providing an overview of recent advances in managing patients with IPF and has a particular focus on how to reach a diagnosis, manage comorbidities and lung transplantation, care for the non-pharmacological needs of patients, and address palliative care. As we have known for a long time from the oncology field, patients receiving palliative care need less aggressive care at the end of life (EOL) and have better quality of life. Antifibrotic treatment with pirfenidone and nintedanib was discussed in the March 2014 ^4^ and May 2016 ^5^ issues of this journal and is not the purpose of this review.

## Lung transplantation

Lung transplantation is an important option to improve the survival of eligible patients ^6^ and represents a treatment option for patients who fail to respond to medical treatment and progress to an advanced stage of the disease ^7, 8^. We know that survival after lung transplantation at 5 years is about 50% (47%–53%). Post-transplant survival for interstitial lung disease (ILD) patients is 4.7 years, much lower than for other underlying pre-transplant diseases ^6^ (that is, less than post-transplant survival for patients with chronic obstructive pulmonary disease (COPD) and cystic fibrosis ^9^). While lung transplantation is the only possible therapy in severe IPF, it may be challenged by several complications: (1) infections and neoplasms in native lung ^7, 8^ (in case of single-lung recipients), (2) extra-pulmonary comorbidities exacerbated by the transplant (that is, heart failure, osteoporosis, and so on) ^7^, (3) chronic lung allograft dysfunction (CLAD), and (4) recurrence of the disease in the graft, another rare but observed complication ^10^. CLAD includes both the obstructive pattern (restraining bronchiolitis obliterans syndrome) and less frequently the restrictive pattern ^7^. Size mismatch is a risk factor for the development of airway complications such as fistula, granulation, bronchomalacia, or strictures. Strictures may occur at the site of surgical anastomosis but may also occur distally, isolated, or as part of the so-called vanishing syndrome ^7^.

Therefore, appropriate selection of lung transplant recipients is an important determinant of outcomes. Since lung transplantation presents a significant risk of perioperative morbidity and mortality, it is important to consider the overall sum of contraindications and comorbidities. In the consensus document for the selection of lung transplant candidates, Weill and colleagues summarized timing of referral and listing, relative and absolute indications, and contraindications for lung transplant ^8^. In order to ensure the best outcome, the functional status of IPF patients listed for lung transplant should be maintained as best as possible. For this reason, patients should actively participate in a supervised pulmonary rehabilitation programme ^7^. Although lung transplantation is an effective therapy, less than 20% of patients with IPF receive a lung transplant and this is because of delay in referral to transplant centres and high mortality in transplant list ^11^.

## Medical approach to patients with idiopathic pulmonary fibrosis

We have recently acquired two drugs approved for the treatment of IPF—pirfenidone and nintedanib—that have been shown to slow the loss of lung function. However, both drugs have shown little effect in improving patient symptoms. Because of this, quality of life remains an important unmet medical need, as does the treatment approach for more severe cases. Most of the patients with an advanced stage of the disease often fail in understanding and accepting the disease course variability and experience anxiety as the disease relentlessly progresses ^12^. For these reasons, patients with IPF should be evaluated for eligibility to palliative care in order to manage these symptoms. In addition to symptoms of anxiety about the disease itself, patients with IPF often show associated comorbidities—for example, pulmonary hypertension (PH), gastroesophageal reflux disease (GERD), obstructive sleep apnoea (OSA), COPD, pulmonary embolism, ischaemic heart disease, and lung cancer—which need to be considered and treated and which may significantly modify prognosis.

## Recent advances in idiopathic pulmonary fibrosis treatment follow progress in pathobiology

Although IPF pathobiology is not yet fully understood, knowledge has substantially improved which has shifted the approach to treatment. In 2000, the American Thoracic Society recommended corticosteroids in addition to cytotoxic agents (cyclophosphamide or azathioprine) on the basis of the hypothesis that inflammation preceded fibrosis ^1, 13^. Current hypotheses suggest a pattern of abnormal wound-healing responses driven by ongoing alveolar epithelial microinjuries, which induce a fibrotic environment. It is assumed that the growth factors secreted by the injured epithelial cells and macrophages promote fibroblast recruitment, proliferation, and differentiation into myofibroblasts. This results in myofibroblast foci, the histological hallmark of usual interstitial pneumonia (UIP), in which the excessive collagen production leads to scarring of the lung and progressive destruction of the lung architecture, especially in ageing subjects or in those with genetic predisposition ^5, 14^. Recently, clinical trials have shifted their attention from anti-inflammatory and immunosuppressant drugs to new mechanisms and pathogenetic pathways. Since the 2005 Idiopathic Pulmonary Fibrosis International Group Exploring N-Acetylcysteine I Annual (IFIGENIA) study ^15^, many clinical trials were published on IPF treatment which used, for instance, interferon gamma-1b (INSPIRE) ^16^; prednisone, azathioprine, and *N*-acetylcysteine with an arm using these drugs in combination therapy, an arm with *N*-acetylcysteine alone, and a placebo group (PANTHER-IPF) ^17^; endothelin receptor antagonists (bosentan ^18^ and ambrisentan ^19^); sildenafil, a phosphodiesterase 5 inhibitor ^20^; warfarin versus placebo ^21^; imatinib ^22^; and macitentan ^23^. However, all trials failed their end points. In addition, the PANTHER-IPF trial showed that treatment with prednisone, azathioprine, and *N*-acetylcysteine was harmful compared with placebo ^17^. All trials involved patients with mild to moderate IPF. The only study considering severe IPF—diffusion capacity of the lungs for carbon monoxide of less than 35%—is the one using sildenafil ^20^. In this double-blind, randomized, placebo-controlled trial, the hypothesis was that treatment with sildenafil would improve walk distance, dyspnoea, and quality of life in patients with advanced IPF. This study did not show a benefit for sildenafil for the primary outcome, which was the proportion of patients with an increase in the 6-minute walk distance (6MWD) of 20% or more. Nevertheless, sildenafil-treated patients have some differences favouring sildenafil in some secondary outcomes, including the degree of dyspnoea and quality of life. In the subsequent sub-analysis from Han and colleagues, the aim was to determine whether sildenafil improves 6MWD in subjects with IPF and right ventricular dysfunction ^24^. The conclusion was that sildenafil treatment results in better preservation of exercise capacity (a significant difference in 6MWD of 99 metres was observed between patients with and without right ventricular dysfunction) and also improves quality of life ^24^.

Since 2011, a new interest in antifibrosing drugs has appeared. After Japanese studies ^25, 26^ on pirfenidone (a drug suppressing transforming growth factor-beta and tumour necrosis factor-alpha with a complex and, in part, unknown mechanism of action), the first large international randomized controlled studies were published and nintedanib (a tyrosine kinase inhibitor) was investigated ^27, 28^. Both pirfenidone and nintedanib demonstrated a reduction in IPF progression, measured by the reduction in forced vital capacity (FVC) decrease as the primary end point, which is consistent with a slowing of disease progression ^29, 30^. Nevertheless, these drugs obtained a conditional recommendation by the 2015 update of the 2011 Clinical Practice Guideline ^31^ (there were no data about patients with more severe IPF and with other comorbidities, and the optimal duration of therapy was unknown). Moreover, Nathan and colleagues found significant differences favouring pirfenidone therapy compared with placebo for treatment-emergent all-cause mortality, IPF-related mortality, and treatment-emergent IPF-related mortality ^32^. Indeed, scientific knowledge advances faster than what guidelines can keep up with.

## Management of comorbidities

Patients with IPF often experience comorbidities such as PH, emphysema and COPD, pulmonary infection, lung cancer, GERD, cardiovascular disease (that is, acute coronary disease and deep vein thrombosis), diabetes mellitus, and OSA ^33, 34^. PH—defined as a mean pulmonary artery pressure of more than 25 mm Hg on right heart catheterization (RHC)—may affect patients at the early stage or during the course of the disease. The prevalence of PH ranges from 8% to 50% depending on the population studied (early or advanced IPF) and on how PH is studied (RHC or echocardiogram). PH causes more severe exertional dyspnoea and increases patient 1-year mortality compared with patients without PH. It is also important to detect other comorbidities that may cause or contribute to PH, such as OSA, congestive heart failure, and pulmonary embolism ^35, 36^. Currently, medications approved for the treatment of pulmonary arterial hypertension have not shown utility in the treatment of PH associated with IPF ^18, 19, 23^. In particular, because it seems to worsen the prognosis, the use of ambrisentan is not recommended ^19, 37, 38^.

The incidence of lung cancer ranges from 1% to 48%, and it has been demonstrated that squamous cell carcinoma is the most common cancer type with a significantly worse survival rate ^33^. Therapeutic possibilities should be considered carefully with a detailed risk-benefit analysis. Since surgery may provoke AEs and postoperative morbidity, surgical intervention should be performed in a selected group of patients. In addition, patients with IPF and lung cancer who are not treated surgically should be carefully treated with chemotherapy, and the indication of chemotherapy or radiotherapy (or both) should be evaluated case by case in terms of cost-benefit to the patient, as the same therapies can lead to AEs ^33^.

GERD prevalence is 87–94% and is thought to be involved in pathogenetic mechanisms and to trigger AEs. The use of proton pump inhibitors (PPIs) remains controversial: on the one hand, Lee and colleagues reported that the use of GERD medication (that is, H _2_ blockers or PPIs) in a large cohort of patients with IPF was associated with a lower high-resolution computed tomography (HRCT) fibrosis score and longer survival; on the other hand, PPIs only affect the acidity of the reflux without preventing reflux or microaspiration ^39^. In addition, a recent study by Kreuter and colleagues found that antacid therapy did not improve outcomes in patients with IPF and that it might potentially be associated with an increased risk of infection in patients with an advanced stage of the disease (FVC < 70%) ^40^. The use of antacid therapy should be contextualized according to the clinical features of each patient with IPF ^41^. However, more studies assessing the role of antacid therapy in IPF are needed since neither the efficacy nor the safety of PPIs in IPF can currently be assumed ^42^.

The syndrome of combined pulmonary fibrosis and emphysema (CPFE) is defined by the simultaneous presence of combined emphysema of the upper lobes and fibrosis of the lower lobes on chest computed tomography. It is characterized by sub-normal spirometry with preserved lung volumes, strongly impaired carbon monoxide diffusing capacity of the lung, and hypoxaemia at exercise, high prevalence of PH, and poor survival. Patients with CPFE have a higher frequency of both lung cancer and PH, which worsens their prognosis; there are no currently available therapies for this disease phenotype ^43, 44^. Smoking is the principal risk factor for both emphysema and fibrosis ^33^.

In conclusion, early identification and appropriate treatment of comorbidities are necessary to minimize their impact on the clinical course of IPF and progression of disease ^45^.

## Palliative care and end of life of patients with idiopathic pulmonary fibrosis

Quality of life is compromised by clinical symptoms (cough and dyspnoea) and poor prognosis. Dyspnoea causes social isolation and limitation in daily activities ^46, 47^. Most of the patients with dyspnoea experience depression and anxiety ^48^. Patients with IPF have chronic respiratory disease without disease-reversing treatment options and face progressive decline. Although the care needs of patients with IPF and their caretakers are similar to those of patients with cancer, early referral to palliative care for these patients is most often not considered ^49^.

The World Health Organization definition of palliative care is the following: “it is an approach that improves the quality of life of patients and their families facing the problem associated with life-threatening illness, through the prevention and relief of suffering by means of early identification and impeccable assessment and treatment of pain and other problems, physical, psychosocial and spiritual” ^50^. Originally, palliative care was provided as EOL treatment, but it can also be used and adjusted for every disease stage, and patients with IPF represent a minority group eligible for early referral to palliative care ^3, 51^. Currently, there is little scientific evidence supporting the use of palliative care in IPF and increasing its use could significantly improve patient quality of life.

**Figure 1.**
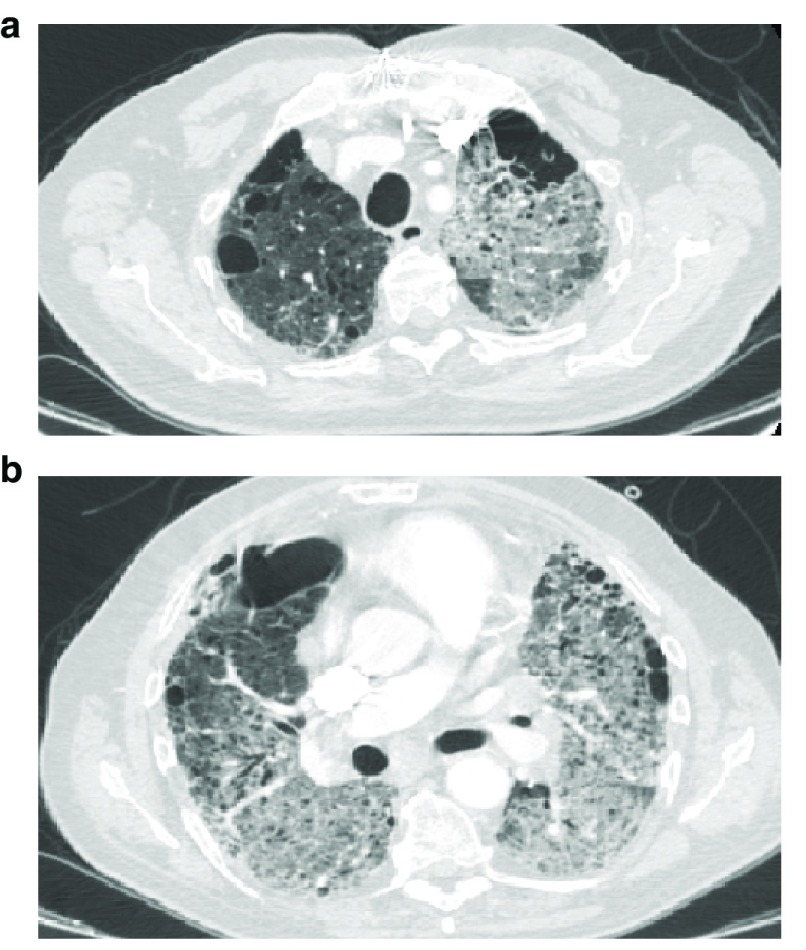
Chest computed tomography showing diffuse ground glass during acute exacerbation of idiopathic pulmonary fibrosis. Bilateral and diffuse radiographic ground glass opacities in a case of acute exacerbation of idiopathic pulmonary fibrosis, **a**) upper lobes and **b**) middle-lower lobes involvement.

Lindell and colleagues conducted a retrospective study demonstrating that palliative care access for patients with IPF is marginal (13.7%) and delayed ^49^. A trial by Higginson and colleagues supports the utility of palliative care in patients with pulmonary but not neoplastic disease ^52^. This study has some limitations due to its short duration and the small population involved. Less attention is given to IPF patients about their prognosis and referral to intensive care than for patients with metastatic cancer ^53^, including patients with very severe oxygen-dependent diseases ^54^. Rajala and colleagues showed that do-not-resuscitate orders and EOL decisions are made late in patients’ lifespan and that life-prolonging therapies are likely to continue until the last days of life ^55^. Death related to IPF is mainly due to respiratory failure caused by either disease progression or AE. According to a consensus perspective statement published in 2016, AE is “an acute, clinically significant deterioration of unidentifiable cause in a patient with underlying IPF” ^56^. Other diagnostic criteria are the occurrence of unexplained worsening of dyspnoea within 30 days, chest HRCT with new bilateral ground glass appearance or consolidation, and the exclusion of alternative causes, including pulmonary infection by endotracheal aspirate or bronchoalveolar lavage (BAL). Clinical features of AE are not easily distinguished from those of other infectious diseases (C-reactive protein level increase and pulmonary infiltrates) ^57^ ( Figure 1). A greater effort should be made (for example, by performing a bronchoscopy with BAL to exclude infectious causes) to reach a diagnosis of certainty before the patient’s condition gets worse. AE is burdened with a high mortality rate. Therapies used are non-invasive ventilation ^58^, parental fluids, antibiotics, high-dose steroids, and morphine. The use of immunosuppressant drugs is debated ^56^.

The most common symptoms experienced in advanced IPF are dyspnoea and cough, which are treated with opioids. Dyspnoea leads to anxiety. Therefore, another important goal to pursue is the treatment of anxiety and depression. These conditions have clinically significant symptoms in up to 30% and 50% of the cases, respectively ^59^. Depressive symptoms are an important predictor of quality of life ^60^. Depression and anxiety treatments have not been studied in IPF, and more and more evidence comes from other populations. The symptoms of depression and anxiety may be alleviated with a strategy of supportive care. These interventions include appropriate analgesia, smoking cessation, oral opiates to control cough and dyspnoea, supplemental oxygen, and pulmonary rehabilitation ^59, 61^. In addition, diminution of the side effects related to antifibrotic medication could improve the anxiety that comes from the chronic illness. Reduced physical activity is common in IPF and other chronic respiratory conditions and is an important predictor of mortality ^62^. Deconditioning is due to multiple reasons, including exertional dyspnoea and symptoms like dry cough that discourage activities. During exercise, the limitations are multifactorial, but a hallmark clinical sign among patients with IPF is the reduction in oxygen saturation caused by a ventilation/perfusion mismatching, oxygen diffusion limitation, and low mixed venous oxygen content ^63^. It should be noted that patients completing short-term interventions see improvements in exercise capacity, dyspnoea, and quality of life. Dowman and colleagues recently demonstrated clinically important improvements in the 6-minute walk test (6MWT), general symptoms, and health-related quality of life following exercise ^64^. Lower baseline 6MWT distance and worse baseline symptoms were associated with greater benefit ^64^. Future studies are needed to define the optimal format and duration of pulmonary rehabilitation for patients with IPF, but given IPF pathophysiology, clinical course, and the robust data of exercise for health benefits, pulmonary rehabilitation supervised programmes should be recommended as standard care for patients with IPF ^59, 63^.

Using drugs to reduce dyspnoea and cough should be another appropriate strategy to reduce anxiety ^55^. The opioid morphine is one of the drugs commonly used with a centrally acting pain-relieving effect. It operates by inhibiting the central perception of dyspnoea, which lowers the feelings of anxiety and fear. The local effect on opioid receptors in the respiratory tract lowers the level of sensory input to the perceptual areas, which directly affects the central perceptual processing and thus the total sensation of dyspnoea ^65^. Kohberg and colleagues showed that the major adverse event was constipation but that respiratory depression, the most feared side effect, was not observed in any of the studies ^65^. It also seemed that central mechanisms for dyspnoea were more important in IPF than local intra-bronchial mechanisms. Finally, morphine seemed to have an effect both at rest and during exercise.

Patients with IPF often spent 15% of their last 6 months of life at the hospital. The majority of them also die there. The most probable reason for this result is the lack of EOL discussions, care plans as well as limited use of palliative care services and home hospice care for non-malignant diseases. These patients need support to deal with their chronic disease and help them face their symptoms. Palliative care would be helpful for psychological support and would bring great comfort to these patients.

## Invasive diagnostic techniques in idiopathic pulmonary fibrosis: focus on cryobiopsy

Another open issue is the time spent to reach final diagnosis. There is often an important time-lag between the appearance of the symptoms and the final diagnosis. Furthermore, although the radiological pattern of UIP is used in the diagnosis of IPF, not all patients with IPF have a UIP pattern on chest computed tomography scan. The typical radiological UIP pattern requires the presence of sub-pleural basal predominance of reticular abnormality and honeycombing with or without traction bronchiectasis. Based on current guidelines, a definite radiological diagnosis of IPF requires the presence of honeycombing, the more specific feature of UIP ^1^. HRCT features of honeycombing are clustered cystic air space, usually of comparable diameters (3–10 mm, occasionally larger) in overlap lines, mainly sub-pleural and characterized by thick and well-defined walls ^1^.

In addition, when the radiological picture is not typical, a histological diagnosis should be considered. Histological UIP pattern is characterized by the following four criteria: evidence of marked fibrosis/architectural distortion, honeycombing in a predominantly sub-pleural/paraseptal distribution, presence of patchy bits of lung parenchyma caused by fibrosis, and presence of fibroblast foci ^1^.

However, owing to disease severity, patient comorbidities, or lack of patient consent, the histological confirmation of IPF through an open lung biopsy is often not possible. Therefore, in some reference centres, a new diagnostic procedure has been employed over the last few years, namely cryobiopsy performed through broncoscopy. This procedure yields larger tissue samples than conventional transbronchial biopsy (TBB), which increases the probability of reaching a diagnosis of ILD (either IPF or non-IPF) in selected patients. Regardless of how the histological confirmation is reached, it should be remembered that the diagnosis of IPF requires the presence of an idiopathic UIP pattern and the exclusion of all known causes (connective tissue disease such as rheumatoid arthritis or scleroderma and drugs-related causes) ^1^.

**Figure 2.**
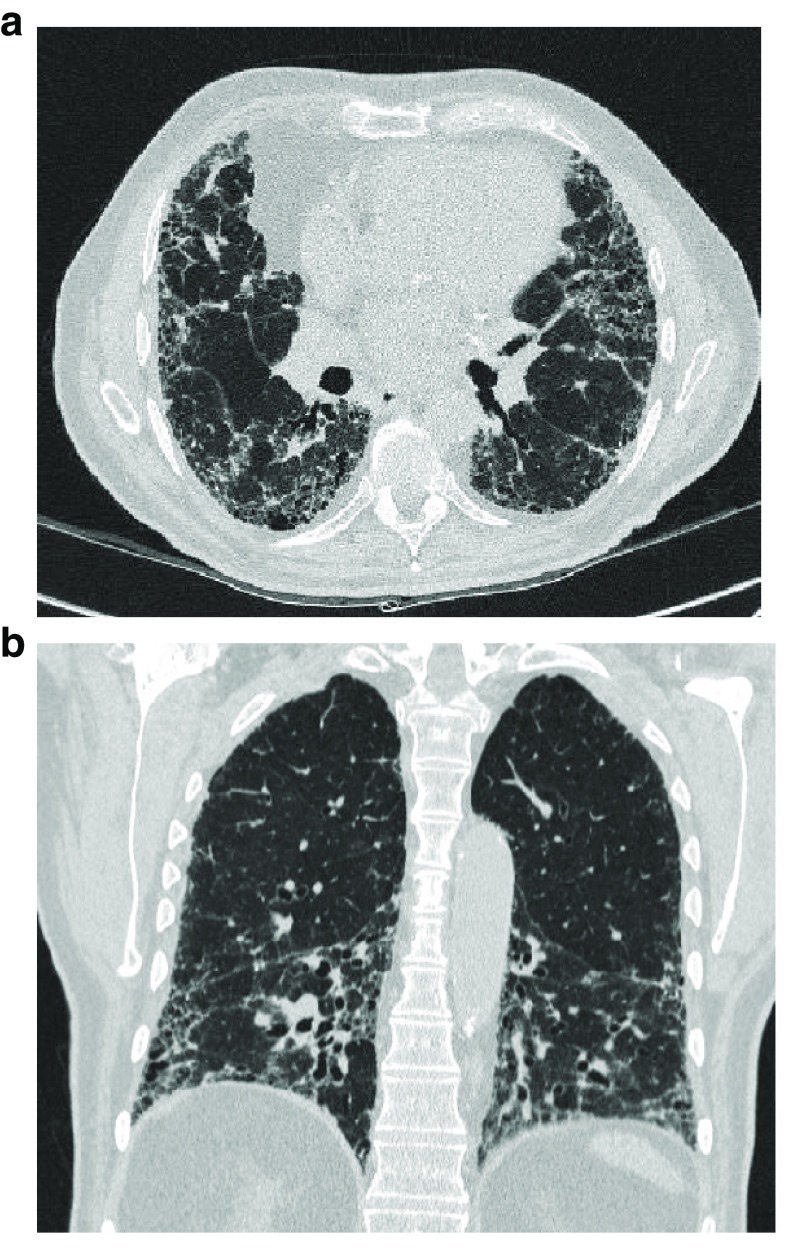
Typical high-resolution computed tomography pattern of usual interstitial pneumonia. **a**) patchy, predominantly peripheral, sub-pleural and bibasal reticular abnormalities, traction bronchiectasis and bronchiolectasis, and irregular septal thickening associated with honey combing, **b**) coronal CT section showing bronchiectasis and bronchiolectasis in the bibasal zones.

**Figure 3.**
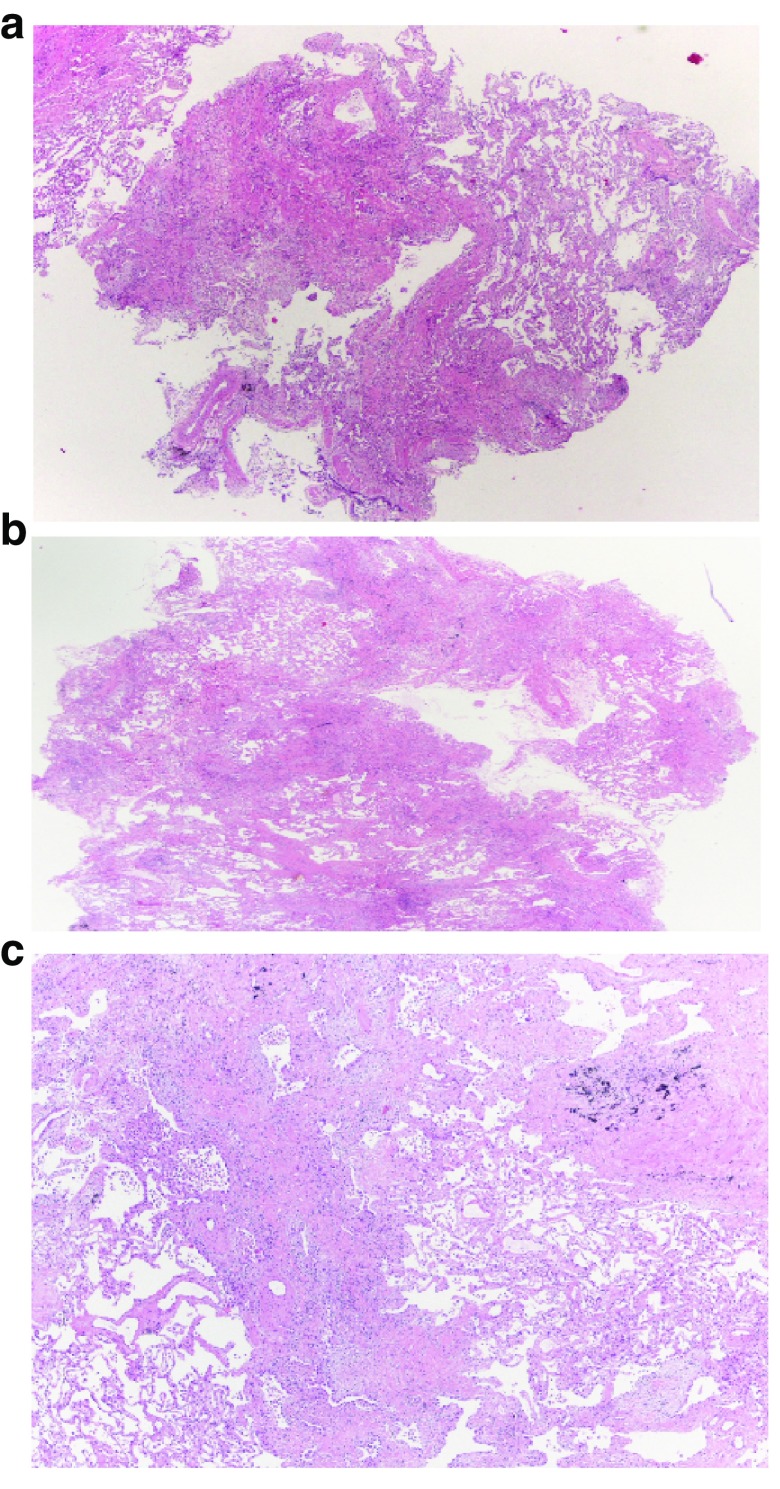
Lung cryobiopsy specimen from a man with a histopathological usual interstitial pneumonia pattern. **a**) and **b**) patchy fibrosing pattern is readily apparent at scanning power microscopy; **c**) numerous fibroblast foci are also readily identifiable (histological section kindly provided by Alberto Cavazza).

Making a diagnosis of IPF requires a multidisciplinary approach integrating clinical profile, radiological appearance, laboratory data and, when those data are inconclusive, invasive procedures to reach histological findings ^1^. Pulmonologists have developed several types of procedures ranging from less invasive ones, like BAL and endobronchial biopsy, to more invasive ones, like TBB with conventional forceps or cryoprobes (c-TBB), transbronchial needle aspiration, endobronchial ultrasound, trans-thoracic biopsies (computed or ultrasound-guided), and medical thoracoscopy, including surgical lung biopsy (SLB). Currently, in most patients with IPF, it is thought that a combination of clinical profile, HRCT scan features, and laboratory tests may lead to satisfactory diagnostic results ^66^ ( Figure 2). Given the new treatment possibilities, it is important to carefully weigh the risks and benefits of the diagnostic tools. Genetic markers—like telomerase reverse transcriptase (TERT) mutations, Mucin 5B (MUC5B) polymorphisms, TOLLIP locus conferring susceptibility, and genes encoding surfactant protein—have been detected in familial and also sporadic cases of IPF ^14, 67^. These markers are present in small numbers of patients and their use is not yet consolidated. Diagnosis may also be reached by using clinical evolution such as functional decline, laboratory tests, or imaging features changing during follow-up which may clarify or modify the previous diagnosis ^68^. Although BAL helps in the diagnostic process and allows clinicians to recognize inflammatory patterns narrowing the differential diagnosis and excluding other possible causes of ILD, it is not specific. TBB still has a controversial role: Berbescu and colleagues demonstrated that UIP hallmarks (patchy fibrosis, honeycombing, and fibroblastic foci) may be identified in TBB samples ^69^. TBB usefulness was confirmed by Tomassetti and colleagues, but the study showed that the predominant histopathological pattern in IPF was the indeterminate pattern ^70^. Therefore, the indeterminate pattern associated with consistent clinical and radiological findings helped in establishing a final diagnosis of IPF. Because of the small size of the biopsy samples, TBB possesses high specificity but low sensibility. More recently, c-TBB has been introduced to obtain larger samples of lung tissue ^71^. The samples obtained usually have a size of between 40 and 50 mm ^2^ and a maximum diameter of between 5 and 7 mm ^71^. Inter-observer variability in UIP pattern recognition is low because samples are retrieved without crash artefacts and contain peripheral structures of the secondary pulmonary lobules ^72^ ( Figure 3). In the near future, immunohistochemical analysis with markers such as heat shock protein 27, MUC5B, β-catenin, and fascin may be carried out on these samples and will provide useful information on IPF background ^67^. The procedure is performed with the aid of fluoroscopy and under deep sedation/general anaesthesia, and patients are intubated with an orotracheal tube or a rigid bronchoscope. Complications are limited and the most common one is pneumothorax (in 8–22% of the cases) ^73^, especially when biopsies are obtained just beneath the pleura. The 30-day mortality is 1.9% ^73^, major bleeding is less than 1%, and other complications (air embolism and AE of fibrotic lung disease) are rarer ^74^. To prevent major bleeding, most bronchoscopists use bronchial blockers (for example, Fogarty balloon) ^72^. The extent of complications depends on the operator’s confidence and expertise with the procedure. This finding is really relevant compared with the rate and type of complications associated with SLB. SLB can lead to prolonged air leak and persisting pain ^75^. SLB is also associated with a significant rate of AEs, which eventually lead to a high mortality rate at 90 days ^76^. Because of this, patient selection is critical, and if patients are carefully selected, morbidity/mortality rates are much lower than in unselected patients ^77^. The value of c-TBB in combination with BAL profiles might be considered in a multidisciplinary diagnostic discussion and thereby allow SLB to be avoided ^78^. The problem remains in the interpretation of the histological specimens obtained by cryobiopsy, which requires experienced pathologists. In real life, it would be best to refer the most complex cases in centres with the best skilled professionals.

## Conclusions and perspectives

In the last three years, the major drug regulatory agencies (the European Medicines Agency and the US Food and Drug Administration) approved the use of two important drugs: pirfenidone and nintedanib. However, IPF management remains difficult because of poor prognosis and because available therapies are not a definite cure but only decrease IPF progression. It is therefore a priority to get a diagnosis as soon as possible in order to gain access to current therapeutic options in early disease stage. We also look forward to seeing the results of many ongoing phase II and III clinical studies ^79^ which evaluate combinations of drugs already approved (pirfenidone and nintedanib) or new drugs that act against matrix deposition.

There is also a growing need for real-life studies that are proven to be useful in clinical practice. Future methodologies should aim at decreasing the use of SLB and developing semi-invasive or invasive procedures like c-TBB together with new diagnostic measures (biomarkers, genetic features, and lavage microbiome) and a more precise interpretation of HRCT scan ^67^. Disease progression is unpredictable and most patients often fail to discern disease course variability. For these reasons, a standardized symptom score could be introduced in order to allow clinicians to assess the level of disease progression and refer patients with IPF to early evaluation for palliative care to improve their outcomes and quality of life ^80^. In recent years, we have seen the development of IPF registries, like the one developed in the US by O’Brien and colleagues ^81^ (IPF-PRO) or the German registry proposed by Behr and colleagues ^82^, to improve understanding in natural history, its impact on patients and current practices in the diagnosis and care of patients with IPF. Last but not least, these registries will remain a repository of samples for future research.

## Abbreviations

6MWD, 6-minute walk distance; 6MWT, 6-minute walk test; AE, acute exacerbation; BAL, bronchoalveolar lavage; CLAD, chronic lung allograft dysfunction; COPD, chronic obstructive pulmonary disease; CPFE, combined pulmonary fibrosis and emphysema; c-TBB, cryo-transbronchial biopsy; EOL, end of life; FVC, forced vital capacity; GERD, gastroesophageal reflux disease; HRCT, high-resolution computed tomography; ILD, interstitial lung disease; IPF, idiopathic pulmonary fibrosis; MUC5B, Mucin 5B; OSA, obstructive sleep apnoea; PH, pulmonary hypertension; PPI, proton pump inhibitor; RHC, right heart catheterization; SLB, surgical lung biopsy; TBB, transbronchial biopsy; UIP, usual interstitial pneumonia.

## References

[1] Raghu G Collard HR Egan JJ: An official ATS/ERS/JRS/ALAT statement: idiopathic pulmonary fibrosis: evidence-based guidelines for diagnosis and management. *Am J Respir Crit Care Med.* 2011;183(6):788–824. 10.1164/rccm.2009-040GL 21471066 PMC5450933

[2] Hutchinson J Fogarty A Hubbard R: Global incidence and mortality of idiopathic pulmonary fibrosis: a systematic review. *Eur Respir J.* 2015: 46(3):795–806. 10.1183/09031936.00185114 25976683

[3] Harari S Madotto F Caminati A: Epidemiology of idiopathic pulmonary fibrosis in Northern Italy. *PLoS One.* 2016;11(2):e0147072. 10.1371/journal.pone.0147072 26841042 PMC4740484

[4] Woodcock HV Maher TM: The treatment of idiopathic pulmonary fibrosis. *F1000Prime Rep.* 2014;6:16. 10.12703/P6-16 24669297 PMC3944742

[5] D’Accord C Maher TM: Recent advances in understanding idiopathic pulmonary fibrosis [version 1; referees: 2 approved]. *F1000 Res.* 2016;5:1–13. pii: F1000 Faculty Rev-1046. 10.12688/f1000research.8209.1 27303645 PMC4890320

[6] Kistler KD Nalysnyk L Rotella P: Lung transplantation in idiopathic pulmonary fibrosis: a systematic review of the literature. *BMC Pulm Med.* 2014;14:139. 10.1186/1471-2466-14-139 25127540 PMC4151866

[7] Brown AW Kaya H Nathan SD: Lung transplantation in IIP: A review. *Respirology.* 2016;21(7):1173–1184. 10.1111/resp.12691 26635297

[8] Weill D Benden C Corris PA: A consensus document for the selection of lung transplant candidates: 2014—An update from the pulmonary transplantation council of the international society for heart and lung transplantation. *J Heart and Lung Transplant.* 2015;34(1):1–15. 10.1016/j.healun.2014.06.014 25085497

[9] Verleden GM Raghu G Meyer KC: A new classification system for chronic lung allograft dysfunction. *J Heart and Lung Transplant.* 2014;33(2):127–133. 10.1016/j.healun.2013.10.022 24374027

[10] Barberis M Harari S Tironi A: Recurrence of primary disease in a single lung transplant recipient. *Transplant Proc.* 1992;24(6):2660–2662. 1465892

[11] Yusen RD Edwards LB Dipchand AI: The registry of the international society for heart and lung transplantation: thirty-third adult lung and heart-lung transplant report-2016; focus theme: primary diagnostic indications for transplant. *J Heart Lung Transplant.* 2016;35(10):1170–1184. 10.1016/j.healun.2016.09.001 27772669

[12] Lindell KO Liang Z Hoffman LA: Palliative care and location of death in decedents with idiopathic pulmonary fibrosis. *Chest.* 2015;147(2):423–429. 10.1378/chest.14-1127 25187973 PMC4314817

[13] American Thoracic Society, European Respiratory Society: American Thoracic Society/European Respiratory Society international multidisciplinary consensus classification of the idiopathic interstitial pneumonias. This joint statement of the American Thoracic Society (ATS), and the European Respiratory Society (ERS) was adopted by the ATS board of directors, June 2001 and by the ERS Executive Committee, June 2001. *Am J Respir Crit Care Med.* 2002;165(2):277–304. 10.1164/ajrccm.165.2.ats01 11790668

[14] Harari S Caminati A: IPF: new insight on pathogenesis and treatment. *Allergy.* 2010;65(5):537–553. 10.1111/j.1398-9995.2009.02305.x 20121758

[15] Demedts M Behr J Buhl R: High-dose acetylcysteine in idiopathic pulmonary fibrosis. *N Engl J Med.* 2005;353(21):2229–2242. 10.1056/NEJMoa042976 16306520

[16] King TE Jr Albera C Bradford WZ: Effect of interferon gamma-1b on survival in patients with idiopathic pulmonary fibrosis (INSPIRE): a multicentre, randomised, placebo controlled trial. *Lancet.* 2009;374(9685):222–228. 10.1016/S0140-6736(09)60551-1 19570573

[17] Idiopathic Pulmonary Fibrosis Clinical Research Network, Raghu G Anstrom KJ: Prednisone, azathioprine, and N-acetylcysteine for pulmonary fibrosis. *N Engl J Med.* 2012;366(21):1968–1977. 10.1056/NEJMoa1113354 22607134 PMC3422642

[18] King TE Jr Brown KK Raghu G: BUILD-3: a randomized, controlled trial of bosentan in idiopathic pulmonary fibrosis. *Am J Respir Crit Care Med.* 2011;184(1):92–99. 10.1164/rccm.201011-1874OC 21474646

[19] Raghu G Behr J Brown KK: Treatment of idiopathic pulmonary fibrosis with ambrisentan: a parallel, randomized trial. *Ann Intern Med.* 2013;158(9):641–649. 10.7326/0003-4819-158-9-201305070-00003 23648946

[20] Idiopathic Pulmonary Fibrosis Clinical Research Network, Zisman DA Schwarz M: A controlled trial of sildenafil in advanced idiopathic pulmonary fibrosis. *N Engl J Med.* 2010;363(7):620–628. 10.1056/NEJMoa1002110 20484178 PMC3587293

[21] Noth I Anstrom KJ Calvert SB: A placebo-controlled randomized trial of warfarin in idiopathic pulmonary fibrosis. *Am J Respir Crit Care Med.* 2012;186(1):88–95. 10.1164/rccm.201202-0314OC 22561965 PMC3400994

[22] Daniels CE Lasky JA Limper AH: Imatinib treatment for idiopathic pulmonary fibrosis: Randomized placebo-controlled trial results. *Am J Respir Crit Care Med.* 2010;181(6):604–610. 10.1164/rccm.200906-0964OC 20007927

[23] Raghu G Million-Rousseau R Morganti A: Macitentan for the treatment of idiopathic pulmonary fibrosis: the randomised controlled MUSIC trial. *Eur Respir J.* 2013;42(6):1622–1632. 10.1183/09031936.00104612 23682110

[24] Han MK Bach DS Hagan PG: Sildenafil preserves exercise capacity in patients with idiopathic pulmonary fibrosis and right-sided ventricular dysfunction. *Chest.* 2013;143(6):1699–1708. 10.1378/chest.12-1594 23732584 PMC3673665

[25] Azuma A Nukiwa T Tsuboi E: Double-blind, placebo-controlled trial of pirfenidone in patients with idiopathic pulmonary fibrosis. *Am J Respir Crit Care Med.* 2005;171(9):1040–1047. 10.1164/rccm.200404-571OC 15665326

[26] Taniguchi H Ebina M Kondoh Y: Pirfenidone in idiopathic pulmonary fibrosis. *Eur Respir J.* 2010;35(4):821–829. 10.1183/09031936.00005209 19996196

[27] Noble PW Albera C Bradford WZ: Pirfenidone in patients with idiopathic pulmonary fibrosis (CAPACITY): two randomised trials. *Lancet.* 2011;377(9779):1760–1769. 10.1016/S0140-6736(11)60405-4 21571362

[28] Richeldi L Costabel U Selman M: Efficacy of a tyrosine kinase inhibitor in idiopathic pulmonary fibrosis. *N Engl J Med.* 2011;365(12):1079–87. 10.1056/NEJMoa1103690 21992121

[29] Richeldi L du Bois RM Raghu G: Efficacy and safety of nintedanib in idiopathic pulmonary fibrosis. *N Engl J Med.* 2014;370(22):2071–2082. 10.1056/NEJMoa1402584 24836310

[30] King TE Jr Bradford WZ Castro-Bernardini S: A phase 3 trial of pirfenidone in patients with idiopathic pulmonary fibrosis. *N Engl J Med.* 2014;370(22):2083–2092. 10.1056/NEJMoa1402582 24836312

[31] Raghu G Rochwerg B Zhang Y: An Official ATS/ERS/JRS/ALAT Clinical Practice Guideline: Treatment of Idiopathic Pulmonary Fibrosis. An Update of the 2011 Clinical Practice Guideline. *Am J Respir Crit Care Med.* 2015;192(2):e3–19. 10.1164/rccm.201506-1063ST 26177183

[32] Nathan SD Albera C Bradford WZ: Effect of pirfenidone on mortality: pooled analyses and meta-analyses of clinical trials in idiopathic pulmonary fibrosis. *Lancet Respir Med.* 2017;5(1):33–41. 10.1016/S2213-2600(16)30326-5 27876247

[33] Suzuki A Kondoh Y: The clinical impact of major comorbidities on idiopathic pulmonary fibrosis. *Respir Investig.* 2017;55(2):94–103. 10.1016/j.resinv.2016.11.004 28274539

[34] Schiza S Mermigkis C Margaritopoulos GA: Idiopathic pulmonary fibrosis and sleep disorders: no longer strangers in the night. *Eur Respir Rev.* 2015;24(136):327–339. 10.1183/16000617.00009114 26028644 PMC9487812

[35] Cerri S Spagnolo P Luppi F: Management of idiopathic pulmonary fibrosis. *Clin Chest Med.* 2012;33(1):85–94. 10.1016/j.ccm.2011.11.005 22365248

[36] Gille T Didier M Boubaya M: Obstructive sleep apnoea and related comorbidities in incident idiopathic pulmonary fibrosis. *Eur Respir J.* 2017;49(6): pii: 1601934. 10.1183/13993003.01934-2016 28596432

[37] Caminati A Cassandro R Harari S: Pulmonary hypertension in chronic interstitial lung diseases. *Eur Respir Rev.* 2013;22(129):292–301. 10.1183/09059180.00002713 23997057 PMC9487353

[38] Harari S Elia D Humbert M: Pulmonary hypertension in parenchymal lung diseases: any future for new therapies? *Chest.* 2017; pii: S0012-3692(17)31077-2. 10.1016/j.chest.2017.06.008 28629920

[39] Lee JS Ryu JH Elicker BM: Gastroesophageal reflux therapy is associated with longer survival in patients with idiopathic pulmonary fibrosis. *Am J Respir Care Med.* 2011;184(12):1390–1394. 10.1164/rccm.201101-0138OC 21700909 PMC3262030

[40] Kreuter M Wuyts W Renzoni E: Antacid therapy and disease outcomes in idiopathic pulmonary fibrosis: a pooled analysis. *Lancet Respir Med.* 2016;4(5):381–389. 10.1016/S2213-2600(16)00067-9 27050871

[41] Johannson KA Strâmbu I Ravaglia C: Antacid therapy in idiopathic pulmonary fibrosis: more questions than answers? *Lancet Respir Med.* 2017;5(7):591–598. 10.1016/S2213-2600(17)30219-9 28664861

[42] https://clinicaltrials.gov/ct2/show/NCT02085018?term=gastroesophageal+reflux&cond=IPF&rank=3.

[43] Mejia M Carrillo G Rojas-Serrano J: Idiopathic pulmonary fibrosis and emphysema: decreased survival associated with severe pulmonary arterial hypertension. *Chest.* 2009;136(1):10–15. 10.1378/chest.08-2306 19225068

[44] Cottin V Nunes H Brillet PY: Combined pulmonary fibrosis and emphysema: a distinct underrecognised entity. *Eur Respir J.* 2005;26(4):586–593. 10.1183/09031936.05.00021005 16204587

[45] Raghu G Amatto VC Behr J: Comorbidities in idiopathic pulmonary fibrosis patients: a systematic literature review. *Eur Respir J.* 2015;46(4):1113–1130. 10.1183/13993003.02316-2014 26424523

[46] Swigris JJ Kuschner WG Jacobs SS: Health-related quality of life in patients with idiopathic pulmonary fibrosis: a systematic review. *Thorax.* 2005;60(7):588–594. 10.1136/thx.2004.035220 15994268 PMC1747452

[47] Lubin M Chen H Elicker B: A comparison of health-related quality of life in idiopathic pulmonary fibrosis and chronic hypersensitivity pneumonitis. *Chest.* 2014;145(6):1333–1338. 10.1378/chest.13-1984 24458311 PMC4042514

[48] Holland AE Fiore JF Jr Bell EC: Dyspnoea and comorbidity contribute to anxiety and depression in interstitial lung disease. *Respirology.* 2014;19(8):1215–1221. 10.1111/resp.12360 25112470

[49] Lindell KO Kavalieratos D Gibson KF: The palliative care needs of patients with idiopathic pulmonary fibrosis: a qualitative study of patients and family caregivers. *Heart Lung.* 2017;46(1):24–29. 10.1016/j.hrtlng.2016.10.002 27871724 PMC5485906

[50] World Health Organization: WHO definition of alliative care—[Internet]. Reference Source

[51] Lanken PN Terry PB DeLisser DM: An Official American Thoracic Society Clinical Policy Statement: palliative care for patients with respiratory diseases and critical illnesses. *Am J Respir Crit Care Med.* 2008;177(8):912–927. 10.1164/rccm.200605-587ST 18390964

[52] Higginson IJ Bausewein C Reilly CC: An integrated palliative and respiratory care service for patients with advanced disease and refractory breathlessness: a randomised controlled trial. *Lancet Respir Med.* 2014;2(12):979–987. 10.1016/S2213-2600(14)70226-7 25465642

[53] Brown CE Engelberg RA Nielsen EL: Palliative Care for Patients Dying in the Intensive Care Unit with Chronic Lung Disease Compared with Metastatic Cancer. *Ann Am Thorac Soc.* 2016;13(5):684–689. 10.1513/AnnalsATS.201510-667OC 26784137 PMC5018894

[54] Ahmadi Z Wysham NG Lundström S: End-of-life care in oxygen-dependent ILD compared with lung cancer: a national population-based study. *Thorax.* 2016;71(6):510–516. 10.1136/thoraxjnl-2015-207439 26865603

[55] Rajala K Lehto JT Saarinen M: End-of-life care of patients with idiopathic pulmonary fibrosis. *BMC Palliat Care.* 2016;15(1):85. 10.1186/s12904-016-0158-8 27729035 PMC5059981

[56] Collard HR Ryerson CJ Corte TJ: Acute Exacerbation of Idiopathic Pulmonary Fibrosis. An International Working Group Report. *Am J Respir Crit Care Med.* 2016;194(3):265–275. 10.1164/rccm.201604-0801CI 27299520

[57] Caminati A Harari S: IPF: New insight in diagnosis and prognosis. *Respir Med.* 2010;104 Suppl 1:S2–S10. 10.1016/j.rmed.2010.03.012 20639137

[58] Aliberti S Messinesi G Gamberini S: Non-invasive mechanical ventilation in patients with diffuse interstitial lung diseases. *BMC Pulm Med.* 2014;14:194. 10.1186/1471-2466-14-194 25476922 PMC4269964

[59] Fulton BG Ryerson CJ: Managing comorbidities in idiopathic pulmonary fibrosis. *Int J Gen Med.* 2015;8:309–318. 10.2147/IJGM.S74880 26451121 PMC4590408

[60] Akhtar AA Ali MA Smith RP: Depression in patients with idiopathic pulmonary fibrosis. *Chron Respir Dis.* 2013;10(3):127–133. 10.1177/1479972313493098 23897928

[61] De Vries J Kessels BL Drent M: Quality of life of idiopathic pulmonary fibrosis patients. *Eur Respir J.* 2001;17(5):954–961. 11488332 10.1183/09031936.01.17509540

[62] Spruit MA Singh SJ Garvey C: An official American Thoracic Society/European Respiratory Society statement: key concepts and advances in pulmonary rehabilitation. *Am J Respir Crit Care Med.* 2013;188(8):e13–64. 10.1164/rccm.201309-1634ST 24127811

[63] Vainsheilboim B: Exercise training in idiopathic pulmonary fibrosis: is it of benefit? *Breathe (Sheff).* 2016;12(2):130–138. 10.1183/20734735.006916 27408631 PMC4933618

[64] Dowman LM McDonald CF Hill CJ: The evidence of benefits of exercise training in interstitial lung disease: a randomised controlled trial. *Thorax.* 2017;72(7):610–619. 10.1136/thoraxjnl-2016-208638 28213592

[65] Kohberg C Andersen CU Bendstrup E: Opioids: an unexplored option for treatment of dyspnea in IPF. *Eur Clin Respir J.* 2016;3:30629. 10.3402/ecrj.v3.30629 26969472 PMC4788766

[66] Jacob J Hansell DM: HRCT of fibrosing lung disease. *Respirology.* 2015;20(6):859–872. 10.1111/resp.12531 25900734

[67] Hambly N Shimbori C Klob M: Molecular classification of idiopathic pulmonary fibrosis: personalized medicine, genetics and biomarkers. *Respirology.* 2015;20(7):1010–1022. 10.1111/resp.12569 26109466

[68] Travis WD Costabel U Hansell DM: An official American Thoracic Society/European Respiratory Society statement: Update of the international multidisciplinary classification of the idiopathic interstitial pneumonias. *Am J Respir Crit Care Med.* 2013;188(6):733–748. 10.1164/rccm.201308-1483ST 24032382 PMC5803655

[69] Berbescu EA Katzenstein AL Snow JL: Transbronchial biopsy in usual interstitial pneumonia. *Chest.* 2006;129(5):1126–1131. 10.1378/chest.129.5.1126 16685001 PMC2094131

[70] Tomassetti S Cavazza A Colby TV: Transbronchial biopsy is useful in predicting UIP pattern. *Respir Res.* 2012;13:96. 10.1186/1465-9921-13-96 23107232 PMC3499172

[71] Casoni GL Tomassetti S Cavazza A: Transbronchial lung cryobiopsy in the diagnosis of fibrotic interstitial lung diseases. *PLoS One.* 2014;9(2):e86716. 10.1371/journal.pone.0086716 24586252 PMC3938401

[72] Poletti V Ravaglia C Gurioli C: Invasive diagnostic techniques in idiopathic interstitial pneumonias. *Respirology.* 2016;21(1):44–50. 10.1111/resp.12694 26682637

[73] Hagmeyer L Theegarten D Treml M: Validation of transbronchial cryobiopsy in interstitial lung disease - interim analysis of a prospective trial and critical review of the literature. *Sarcoidosis Vasc Diffuse Lung Dis.* 2016;33(1):2–9. 27055830

[74] Ravaglia C Bonifazi M Wells AU: Safety and Diagnostic Yield of Transbronchial Lung Cryobiopsy in Diffuse Parenchymal Lung Diseases: A Comparative Study versus Video-Assisted Thoracoscopic Lung Biopsy and a Systematic Review of the Literature. *Respiration.* 2016;91(3):215–227. 10.1159/000444089 26926876

[75] Han Q Luo Q Xie JX: Diagnostic yield and postoperative mortality associated with surgical lung biopsy for evaluation of interstitial lung diseases: A systematic review and meta-analysis. *J Thorac Cardiovasc Surg.* 2015;149(5):1394–401.e1. 10.1016/j.jtcvs.2014.12.057 25648484

[76] Kondoh Y Taniguchi H Kitaichi M: Acute exacerbation of interstitial pneumonia following surgical lung biopsy. *Respir Med.* 2006;100(10):1753–1759. 10.1016/j.rmed.2006.02.002 16584880

[77] Hutchinson JP Fogarty AW McKeever TM: In-Hospital Mortality after Surgical Lung Biopsy for Interstitial Lung Disease in the United States. 2000 to 2011. *Am J Respir Crit Care Med.* 2016;193(10):1161–1167. 10.1164/rccm.201508-1632OC 26646481

[78] Tomassetti S Wells AU Costabel U: Bronchoscopic Lung Cryobiopsy Increases Diagnostic Confidence in the Multidisciplinary Diagnosis of Idiopathic Pulmonary Fibrosis. *Am J Respir Crit Care Med.* 2016;193(7):745–752. 10.1164/rccm.201504-0711OC 26562389

[79] https://clinicaltrials.gov/ct2/results?cond=IPF&term=&cntry1=&state1=&SearchAll=Search+all+studies&recrs.

[80] Wells AU Costabel U Poletti V: Challenges in IPF diagnosis, current management and future perspectives. *Sarcoidosis Vasc Diffuse Lung Dis.* 2015;32 Suppl 1:28–35. 26237442

[81] O’Brien EC Durheim MT Gamerman V: Rationale for and design of the Idiopathic Pulmonary Fibrosis-PRospective Outcomes (IPF-PRO) registry. *BMJ Open Respir Res.* 2016;3(1):e000108. 10.1136/bmjresp-2015-000108 26835134 PMC4716211

[82] Behr J Kreuter M Hoeper MM: Management of patients with idiopathic pulmonary fibrosis in clinical practice: the INSIGHTS-IPF registry. *Eur Respir J.* 2015;46(1):186–96. 10.1183/09031936.00217614 25837040 PMC4486374

